# Current Practice in Untargeted Human Milk Metabolomics

**DOI:** 10.3390/metabo10020043

**Published:** 2020-01-22

**Authors:** Isabel Ten-Doménech, Victoria Ramos-Garcia, José David Piñeiro-Ramos, María Gormaz, Anna Parra-Llorca, Máximo Vento, Julia Kuligowski, Guillermo Quintás

**Affiliations:** 1 Neonatal Research Unit, Health Research Institute La Fe, Avenida Fernando Abril Martorell 106, 46026 Valencia, Spain; isabel_ten@iislafe.es (I.T.-D.); victoria_ramos@iislafe.es (V.R.-G.); jose_pineiro@iislafe.es (J.D.P.-R.); gormaz_mar@gva.es (M.G.); annaparrallorca@gmail.com (A.P.-L.); maximo.vento@uv.es (M.V.); 2 Division of Neonatology, University & Polytechnic Hospital La Fe, Avenida Fernando Abril Martorell 106, 46026 Valencia, Spain; 3 Health and Biomedicine, Leitat Technological Center, Carrer de la Innovació, 2, 08225 Terrassa, Spain; gquintas@leitat.org; 4 Unidad Analítica, Health Research Institute La Fe, Avenida Fernando Abril Martorell 106, 46026 Valencia, Spain

**Keywords:** human milk, metabolome, sampling, extraction, liquid chromatography–mass spectrometry (LC-MS), nuclear magnetic resonance (NMR), gas chromatography–mass spectrometry (GC-MS), capillary electrophoresis—mass spectrometry (CE-MS)

## Abstract

Human milk (HM) is considered the gold standard for infant nutrition. HM contains macro- and micronutrients, as well as a range of bioactive compounds (hormones, growth factors, cell debris, etc.). The analysis of the complex and dynamic composition of HM has been a permanent challenge for researchers. The use of novel, cutting-edge techniques involving different metabolomics platforms has permitted to expand knowledge on the variable composition of HM. This review aims to present the state-of-the-art in untargeted metabolomic studies of HM, with emphasis on sampling, extraction and analysis steps. Workflows available from the literature have been critically revised and compared, including a comprehensive assessment of the achievable metabolome coverage. Based on the scientific evidence available, recommendations for future untargeted HM metabolomics studies are included.

## 1. Introduction

Human milk (HM) has been markedly established as the optimal way of providing infants with the necessary nutrients and bioactive factors for their early development. Many health associations and organisms, including World Health Organization, recommend exclusive breastfeeding for the first six months of life [1]. Health benefits of HM for infants include reduced mortality and morbidity, including sepsis, respiratory diseases, otitis media, gastroenteritis, and urinary tract infections, among others [2]. In addition, studies reporting on long-term benefits of HM consumption such as lower risk of suffering from type 1 diabetes and inflammatory bowel disease or overweight in adulthood emerged [3]. HM may also be associated with a slightly improved neurological outcome as cohort studies report [4], especially in preterm infants [5], although potential confounders must be accounted for [6]. 

HM composition is dynamic and influenced by several factors including genetics, gestational and infant’s age, circadian rhythm, maternal nutrition, or ethnicity. It provides a series of nutrients such as lipids, proteins, carbohydrates, and vitamins, jointly with a number of bioactive factors that contribute to several physiological activities in the newborn infant as well as to short- and long-term outcomes [7,8]. Living cells including stem cells, hormones, growth factors, enzymes, microbiota, and even genetic material are part of this vast array of HM components with impact in early development, particularly the immune system [9]. In addition, HM appears to be one of the richest sources of microRNAs [10]. On the other hand, because of the maternal environmental exposure and lifestyle, the presence of some contaminants such as persistent organic pollutants or pharmacologically active substances in HM has been described [11,12]. 

Due to its complex composition, the analysis of HM is not straightforward. While the advent of “omics” approaches has offered valuable insights into the composition of this unique biofluid, untargeted metabolomic and lipidomic studies have only recently been applied to HM [13]. The comprehensive study of the HM metabolome, which includes the intermediate and end products of metabolism, can shed light on maternal status or phenotype [14,15]. The generation, analysis, and integration of large and complex data sets obtained in metabolomic studies go hand in hand with the following challenges: (i) the intrinsic complexity of the sample: a rich variety of jointly present, structurally heterogeneous compounds at concentrations that strongly vary covering several orders of magnitude; (ii) pre-analytical steps related to sampling, storage, and pre-processing (e.g., extraction, clean-up); and (iii) the diversity of platforms currently available including nuclear magnetic resonance (NMR), as well as gas chromatography (GC), liquid chromatography (LC), and capillary electrophoresis (CE) coupled to mass spectrometry (MS). The analysis of the HM metabolome has been approached employing a variety of extraction and analytical techniques to respond to a spectrum of clinically relevant questions. Several studies have compared HM metabolome with formula milk [13,16,17,18,19,20] or with milk from other mammalian species including monkey [21], donkey [17], and cow [18], whereas others have made efforts in defining the metabolome of preterm milk [13,16,22,23,24,25,26] and the evaluation of the HM metabolome during the course of lactation [15,23,27,28,29,30]. Furthermore, the influence of maternal diet [14,15,31], phenotype [14,32], obesity [30], or atopy status [33], as well as geographical location [33,34], time of the day [29,35], chemotherapy [36], or preeclampsia during pregnancy [31] on the HM metabolome have been reported.

**Figure 1:**
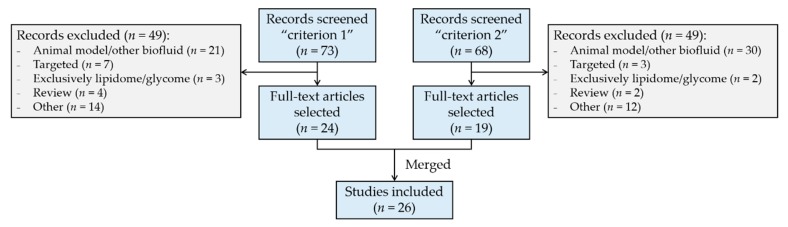
Flow diagram of literature selection and review process. Search “criterion 1”: term (“human milk” OR “breast milk”), AND “metabolom*”, AND “infant”; only articles. Search “criterion 2”: term (“human milk” OR “breast milk”), AND “metabolom*”, AND (“GC” OR “LC” OR “NMR” OR “CE”); only articles. Web of Science database was employed for literature search.

Recent review articles that address the HM metabolome or lipidome [12,37,38,39,40,41] are available. For information on the compounds and compound families typically found in HM and their function the reader is referred to [37,38,39,40]. Readers with a particular interest in HM lipidomics are referred to a recent compilation study [41]. Technical aspects of HM analysis when performing metabolomics studies in HM have been recently described [12]. This review article gathers recent literature available on metabolomic analysis of HM, particularly focusing on untargeted approaches as indicated in Figure 1, to provide an up-to-date overview of the key factors that may influence HM metabolome coverage. Based on the information provided within the available literature, recommendations to guide study design and analytical method development of untargeted HM metabolomics assays were developed.

## 2. Considerations Regarding the Study Design

HM is a biofluid characterized by a dynamically varying composition according to several factors including lactation time, time of the day, throughout each feed, maternal status, and the environmental exposure. Although compositional variations have been mainly studied regarding the protein content of HM [42], changes of other compound classes such as fat or vitamins have been also reported [43,44]. Considering the intrinsic variability of HM, the complexity of obtaining representative HM samples is not negligible. Sources of variation related to sample manipulation and compositional variation can be minimized using standard operational procedures (SOPs). SOPs are fundamental to maintain quality assurance (QA) and quality control (QC) process and facilitate repeatable and reproducible research within and across laboratories. However, biologically meaningful results across studies will only be obtained if several key factors during the sample collection process are successfully controlled. This is of special importance in untargeted approaches, where the interpretation of results is especially challenging, and confounding factors introduced by a non-exhaustive sampling protocol can be wrongly attributed to differences between subjects of a studied population. Conversely, biologically meaningful information can be missed or remain unnoticed due to unwanted bias introduced during sample collection.

**Figure 2:**
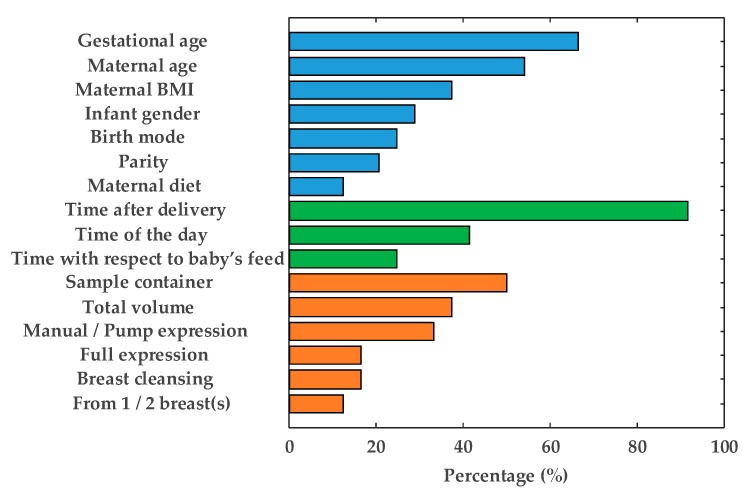
Reporting frequency of factors relevant to the human milk (HM) sampling process: Maternal-infant-related factors (blue bars), time-related factors (green bars), and HM collection-related factors (orange bars). Note: BMI = body mass index.

The information regarding study design provided in HM metabolomics studies varies considerably [13,14,15,16,17,18,19,20,21,22,23,24,26,27,28,29,30,31,32,34,35,36,45,46,47] as shown in Figure 2. Repeatedly reported factors have been grouped into three categories and are discussed in detail in the following sections: (1) maternal-infant-related factors (blue bars), (2) time-related factors (green bars), and (3) HM collection-related factors (orange bars). It should be noted that, although the importance of each factor might vary with the scientific question of each study, the authors encourage (i) the use of SOPs employed during sample collection to assure homogenous and representative sampling and (ii) the reporting of all documented factors in order to enhance comparability between results of metabolomic studies on HM. In case of HM, samples are typically collected, handled and sometimes temporary stored and transported by the mothers and not, such as it is the case for other biofluids (e.g., plasma or serum), by health professionals. During study design it is therefore very important to assure that mothers receive detailed instructions and/or training for the correct handling of collected samples. In addition, one should keep in mind that sampling protocols should neither interfere with infant feeding nor negatively impact on the mother-baby bonding. Hence, the collection of transitional and mature milk is usually preferred over colostrum, especially in studies involving mothers of preterm infants, where colostrum is usually kept exclusively for the infant’s supply.

### 2.1. Maternal-Infant-Related Factors

In HM metabolomic studies, gestational age is frequently reported (see Figure 2), although the impact of this factor on the HM metabolome has not been fully characterized. Studies focused on preterm milk showed that, analogously to full-term milk, its composition is dynamic throughout the first month of lactation [13,16,22]. However, after 5–7 weeks, metabolite composition of HM from mothers of preterm infants resembled that collected from mothers of full-term infants [23]. On the other hand, Marincola et al. [13] observed that HM from mothers of early preterm infants (26 weeks of gestation) differentiated from milk samples from term infants. However, the low number of samples involved in the study (*n* = 20 and *n* = 3 mothers of preterm and term infants, respectively) hindered the assessment of the statistical significance of the impact of gestational age on the milk metabolite composition. Sundekilde et al. [23] carried out a longitudinal study on milk from mothers of preterm and full-term infants covering similar lactation periods (3–14 weeks and 3–26 weeks after birth, respectively) and showed that some metabolites were present at significantly different levels in full-term milk compared to preterm milk. On the contrary, Longini et al. [16] did not observe significant differences between preterm and full-term milk within the first week after delivery, only being able to discriminate milk samples from early preterm infants (<29 weeks of gestation). It is worth noting that the effect of gestational age on the HM metabolome has been mainly studied employing NMR platforms [13,16,22,23], in which metabolite coverage is limited (see Figure 5) and some metabolite classes (e.g., lipids) are barely accessible. For this reason, and in order to further evaluate the impact of gestational age on the milk metabolome, we warrant more comprehensive metabolomic studies employing complementary analytical platforms.

Other potentially relevant, miscellaneous information about the studied population of mother-infant pairs such as infant gender, parity, and birth mode, have been frequently reported in metabolomic studies (see Figure 2), although these characteristics often remain in the background since the studies focus on other aspects. The influence of these factors on the metabolite composition of HM has not been elucidated yet, and this might be addressed in forthcoming studies. An additional factor that is not typically reported in HM metabolomics studies is maternal secretor status. Significant differences in the oligosaccharides profile of milk between so-called secretors (Se^+^), which are those mothers that provide a functional *FUT2* gene, and non-secretors (Se^−^) have been reported [48]. Secretor status is mainly established based on the presence (Se^+^) or absence (Se^−^) of 2’-fucosyllactose, with a prevalence rate of approximately 80% of secretors over non-secretors [14,22,23,26,32,49]. Maternal secretor status is therefore usually determined *a posteriori* during data processing and analysis. Oligosaccharides are polar compounds that are present at concentrations in the mM range that will likely be preserved during sample extraction procedures employed for metabolomics studies. As their presence/absence might potentially affect clustering of milk based on maternal secretor status [24], to provide this information, when available, might be of interest.

### 2.2. Time-Related Factors

HM undergoes significant changes over time, having established three differentiated lactation stages: colostrum, transitional milk and mature milk. As can be seen in Figure 2, lactation time is reported in the vast majority of metabolomic studies. In particular, several studies have focused on the HM metabolome throughout lactation [15,23,27,28,29,30], all of them concluding that significant differences in the metabolic profile over time exist. Therefore, it seems reasonable to report this factor. On the other hand, although it has been demonstrated that diurnal variation affects HM fat content [50], its effect on the overall metabolite composition is a controversial issue which has not yet been adequately addressed in the available literature. Whereas no significant changes in some lipids and small polar metabolites have been observed [29,35], differences in some micronutrients (e.g., vitamins) could be evidenced [44]. The use of a pool of a 24-h expression of HM should compensate for changes due to diurnal variation, thus, obtaining more representative samples [13,24,25] in longitudinal studies. However, the drawback is that this practice is incompatible with breastfeeding of the infant, which, in turn, might raise severe ethical concerns. A feasible compromise for ameliorating diurnal variations is the use of pooled morning and evening samples [29,35].

Regarding time of collection with respect to baby’s feed, the influence of this variable has not been studied to date, but given the differences found between fore- and hindmilk [51], it seems reasonable to assume that this factor might be potentially relevant.

### 2.3. HM Collection-Related Factors

Any uncontrolled variable within an experiment can result in a potential source of bias. In this sense, although less attention has been payed to other factors related to the expression and storage of HM (see Figure 2, orange bars), they may be relevant to the outcomes of metabolomic studies. As can be seen, the type of sample container is indicated in 50% of the studies, whereas other specifications regarding HM expression are included scarcely. The latter factor deserves some special attention, since differences in the milk fat content between foremilk (initial milk of a feed) and hindmilk (last milk of a feed) have been reported [51]. Therefore, full expression of breast(s) is desirable in order to obtain a representative HM aliquot [52]. The influence of all other factors, to date, remains unstudied.

### 2.4. Pasteurization and Storage

HM banks rely on stringent protocols in which pasteurization, indispensable for minimizing the potential to transmit infectious agents, as well as freezing and long-term storage procedures are established. The pasteurization process affects some of the nutritional and biological properties of HM [53,54,55]. In this review, three studies that use milk from HM banks are included [16,20,23], but only one specifies whether or not HM has undergone pasteurization [23]. Variability of the metabolite profile of HM caused by pasteurization has not been comprehensively explored to date. Future studies focused on the systematic exploration of the effect of thermal treatment on HM are warranted.

HM is usually stored frozen employing −20 °C and −80 °C for short- and long-term storage, respectively. However, duration of storage and the effect of repeated freeze-thaw cycles are identified as additional factors with potential impact on HM composition that are missing in most published studies. In lipidomic studies, the integrity of HM samples is preserved by subjecting HM to inactivation of endogenous enzymes such as lipases in order to minimize lipolysis and lipogenesis. In this sense, immediate storage at −80 °C is advisable [41]. Particularly for metabolite composition analysis, storage at −80 °C is widespread [13,15,18,19,24,25,26,28,31,45,46], sometimes with a prior short-term storage at −20 °C [14,21,22,27,29,33,34]. Wu et al. [29] investigated the effect of storage conditions by keeping samples for different times at −20 °C and then transferring them to −80 °C versus storing samples directly at −80 °C. Variations in duration of storage at −20 °C versus −80 °C showed no detectable effect on the metabolites considered (e.g., lactose and other carbohydrates, choline and its derivatives, and a variety of amino acids) by visual inspection of sample clusters in principal component analysis scores plots. However, analysis of variance evidenced differences in butyrate, caprate, and acetate contents. However, time of storage considered in this study was limited to two weeks, which is not representative for conditions employed in clinical studies or standard home routines. It is therefore clear that further studies are required in this regard.

On the other hand, HM employed for research studies is typically stored in small aliquots. When working with raw milk, this procedure might introduce bias due to phase separation prior to the preparation of the aliquots. Hence, the homogenization of HM with a disruptor, resulting in a stable emulsion with reduced size of milk fat globules [56], as employed prior to the quantitation of macronutrients with HM analyzers, might be advisable.

## 3. Metabolite Extraction from HM

**Figure 3:**
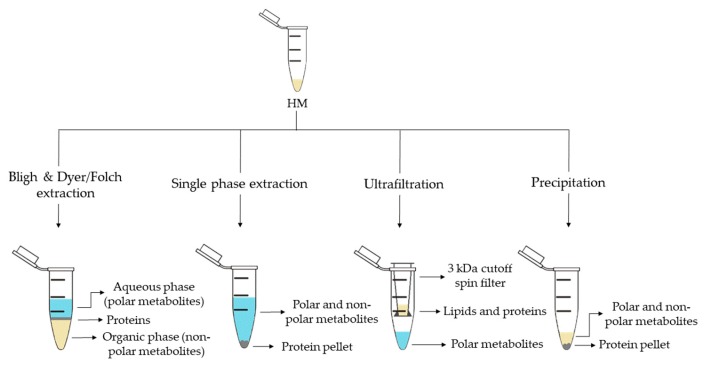
Sample preparation approaches employed in human milk (HM) metabolomics.

For metabolite extraction from HM, an array of methods has been reported. An overview of the employed approaches is shown in Figure 3. The selection of the extraction method is conditioned by the study objective and the subsequent analysis method. As in other untargeted metabolomics workflows, for HM metabolomics, the selected sample preparation approach should enable a high degree of metabolome coverage while making the sample matrix compatible with the analytical platform. Other considerations might include the available amount of sample volume and the use of one sample extraction procedure for subsequent analysis by multiple, complementary analytical platforms [13,27,28].

Liquid-liquid extraction (LLE) is the classical extraction method employed in metabolomics and lipidomics. This method, developed by Folch et al. [57] in 1957, uses a chloroform-methanol mixture (2:1, *v*/*v*), which results in two differentiate phases: an upper phase containing polar metabolites and a lower phase containing nonpolar metabolites. Subsequently, in 1959 Bligh and Dyer [58] developed a modified method using a miscible chloroform-methanol-water mixture and later separated into two phases by adding chloroform or water. Both approaches enable the separation of polar and nonpolar metabolites, thus, allowing the analysis of a wide range of metabolites and making them compatible with several analytical platforms. While the use of Bligh and Dyer LLE is widely extended for HM metabolomics studies (see Table 1) [13,16,17,18,19,24,25,29,32], only Andreas et al. [28] used a modified Folch extraction protocol for processing HM samples.

Methyl tert-butyl ether (MTBE) in combination with methanol has recently been proposed for single-phase extraction [27]. MTBE is a nontoxic and noncarcinogenic solvent and it is therefore considered a safe and environmentally friendly alternative to harmful solvents employed in traditional LLE methods, such as chloroform, which is a suspected human carcinogen. In this extraction method, a unique phase containing both, polar and nonpolar metabolites is obtained with a protein pellet at the bottom (see Figure 3). Thus, the simultaneous analysis of lipidome and metabolome in a very small amount of biological sample is achievable. This method has been successfully employed to determine polar metabolites and fatty acids (FAs) in HM by GC-MS [27,28], as well as lipids and polar metabolites by LC-MS [15,27,28], thus, increasing the metabolome coverage by the combined use of complementary analytical platforms. 

Ultrafiltration makes use of centrifugal molecular weight cutoff filters. Different molecular weight cut-off filters are commercially available for this purpose and repeated centrifugation steps might be employed to remove proteins and lipids (see Table 1). Unlike single-phase extraction, ultrafiltration allows to separate polar metabolites from the HM without dilution [14,21,22,29], however this method does not have the capacity to study the global metabolome of HM. At present, this extraction method has only been used in combination with NMR analyses [14,21,22,29].

Precipitation with organic solvents separates the polar and nonpolar metabolites of the proteins that settle at the bottom of the tube which can then be easily removed by centrifugation. This simple method has been employed for the analysis of polar metabolites by GC-MS after derivatization [36] as well as for the analysis of polar and nonpolar metabolites by LC-MS without further pre-processing [18]. Furthermore, this approach has been implemented in more sophisticated workflows as recently shown by Hewelt-Belka et al. [35]. Here, the authors combined LLE and a protein precipitation and solid-phase extraction (SPE) procedure to prepare HM samples, thereby, enabling the detection of high- and low-abundant lipid species (e.g., glycerolipids and phospholipids) in one LC-MS run.

## 4. Analytical Platforms Employed in HM Metabolomics

As reflected in Table 1, the use of all analytical platforms that are commonly employed in untargeted metabolomics studies, such as LC-MS, GC-MS, NMR, and, to a lesser extent, CE-MS, has been reported for performing HM metabolomics. ^1^H-NMR is the most frequently used technique [13,14,16,20,21,22,26,28,29,31,32,33] for both, the analysis of polar and hydrophobic metabolites in HM. ^13^C-NMR has been reported for the detection of triacylglycerols [20]. NMR is a highly reproducible technique that allows a straightforward library matching after spectral alignment, while at the same time supporting structural elucidation of detected metabolites. However, it presents lower sensitivity, and hence, the achievable metabolome coverage is low in comparison to other analytical platforms. 

All other analytical platforms rely on the use of MS detection. LC-MS provides high sensitivity and is characterized by a huge versatility due to the availability of (i) a large selection of chromatographic columns with a variety of stationary phases, that in combination with appropriate mobile phases achieve compound separation based on different retention mechanisms and (ii) an array of different instrumental configurations (i.e. different ion sources and mass analyzers). For example, reversed phase (C8, C18) LC-quadrupole time of flight MS (LC-QTOF-MS) [15,18,27,28,35,46] and LC-Orbitrap-MS [24,25] have been reported for the detection of both, polar and lipidic metabolites in HM; and hydrophilic interaction LC (HILIC)-QTOF-MS for polar metabolite detection [35]. On the other hand, for the successful screening of HM oligosaccharides, a porous graphitic carbon column installed on a LC-triple quadrupole (TQD)-MS instrument was used [24].

GC-MS is the most suitable platform for measuring volatile compounds, while other non-volatile compounds must be derivatized prior to analysis. For HM analysis, methoximation followed by silylation or methylation are commonly employed (see Table 1). The most frequently used column is the DB-5ms column for both polar and FA detection [13,17,18,19], in some cases with an integrated 10 m pre-column (deactivated fused silica) [27,28,36].

Regarding CE-MS, only one study has been reported for polar metabolite detection in HM [28]. CE provides a series of advantages over other techniques, mainly due to the small sample volumes employed and the efficient separation of polar compounds that is difficult to be achieved by LC columns. However, issues with poor reproducibility, matrix effects and sensitivity may be hindering a widely extended use of this technique for the analysis of complex biological samples such as HM.

Due to the diversified composition of HM, no single analytic technique can resolve the entire HM metabolome. Only multiplatform approaches enable a comprehensive characterization providing a high metabolome coverage including polar and nonpolar metabolites present in HM. In this sense, two studies performing a multiplatform approach were found in the literature combining LC-MS and GC-MS [18,27], and only one study that performed analysis using four different techniques (LC-MS, GC-MS, NMR, and CE-MS) was reported [28].

The use of high-end analytical platforms requires the implementation of QA and QC processes to improve data quality, repeatability, and reproducibility, especially in untargeted metabolomics. For practical guidelines on the use of QC measures in untargeted, MS-based assays the reader is referred to [59]. Pooled QC samples are prepared by mixing small aliquots of the study samples, and therefore, they are considered representative in terms of matrix composition and concentration ranges of the metabolites present in the study samples. QC samples are analyzed repeatedly throughout the analytical sequence alongside the study samples. The signal of each feature detected in QC samples can be used to model and correct systematic changes in the instrument response during the analytical sequence. Additionally, the obtained data can be used to perform intra-study reproducibility assessments and to correct for systematic variation across batches. In HM metabolomics, Smilowitz et al. [14], Andreas et al. [28], and Gay et al. [33] used QC samples for NMR studies, while Villaseñor et al. [27], Mung et al. [46], Hewelt-Belka et al. [35], and Alexandre-Gouabau et al. [24,25] used pooled HM samples for QC purposes in LC-MS-based assays. Considering the highly complex sample matrix of HM, the authors strongly recommend the implementation of QC measures, including the analysis of QC samples, to increase reproducibility and facilitate the joint analysis of data from different studies.

## 5. The HM Metabolome: Compound Annotation and Coverage

As in other areas of metabolomic research, compound identification is still a major bottleneck in data analysis and interpretation. The Metabolomics Standards Initiative’s (MSI) defines four levels of metabolite identification, which include: identified metabolites (level 1); putatively annotated compounds (level 2); putatively annotated compound classes (level 3); and unknown compounds (level 4) [60]. Due to the limited availability of pure analytical standards required to reach level 1, biological databanks and spectral databases are the most important resources for metabolite annotation (levels 2 and 3). A large number of databases are available today, providing different levels of information and complementary data on chemical structures, physicochemical properties, biological functions, and pathway mapping of metabolites [61]. The metabolomics community classifies these resources in several categories: (i) chemical databases; (ii) spectral libraries; (iii) pathway databases; (iv) knowledge databases; and (v) references repositories [62].

Regarding HM metabolomics, the most frequently used databases and libraries are: Human Metabolome Database (HMDB) [63], Metabolite and Chemical Entity Database (METLIN) [64], National Institute of Science and Technology (NIST) library, Fiehn RTL Library [65], LipidMAPS Structure Database (LMSD) [66], Milk Metabolome Database (MCDB) [67,68], Kyoto Encyclopedia of Genes and Genomes (KEGG) [69], MycompoundID with the evidence-based metabolome library (EML) [70], Chenomx NMR Suite Profiles and other online university databases, such as CEU-mass mediator [71,72].

Metabolite assignment in NMR spectra has been performed based on literature data and commercial resonance databases, such as Chenomx NMR Suite Profiles. Metabolite annotation was contrasted with in-house libraries containing pure compound spectra. Some of the proposed assignments were confirmed by two-dimensional NMR spectra, such as Correlation Spectroscopy (COSY) [13,29,31,32], Homonuclear Correlation Spectroscopy (TOCSY) [13,31,32,34], Diffusion-Ordered Spectroscopy (DOSY) [32], Heteronuclear Single Quantum Coherence Spectroscopy (HSQC) [32,34], and Heteronuclear Multiple Bond Correlation (HMBC) [32].

In LC-MS and CE-MS-based studies of the HM metabolome, tentative metabolite annotation has been carried out by matching of accurate masses, isotopic profiles, and/or fragmentation patterns to candidate metabolites in online databases such as KEGG, METLIN, LipidMAPS, and HMDB [18,24,25,27,28,35]. In-house built databases generated by the analysis of commercial standards are also commonly employed [24,25]. In GC-MS, retention index (RI) corrections are made by analyzing a fatty acid methyl ester (FAME) mixture standard solution and assigning a match score between the experimental FAME mixture and theoretical RI values based on the values contained in the Fiehn RTL library. Furthermore, metabolites were complementarily annotated by comparing their mass fragmentation patterns with those available in Fiehn RTL and NIST libraries [13,17,18,19,27,28,36].

**Figure 4:**
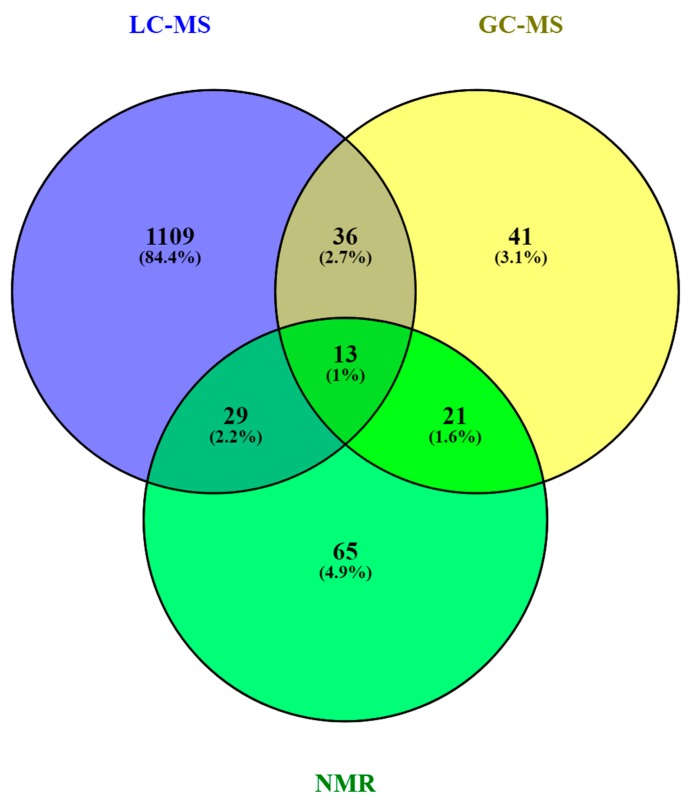
Venn diagram of metabolites reported in human milk (HM) according to the technique in [73]. Note: GC-MS, gas chromatography—mass spectrometry; LC-MS, liquid chromatography—mass spectrometry; NMR, nuclear magnetic resonance.

A comprehensive list of annotated and/or identified metabolites in HM from untargeted metabolomics studies [14,15,17,18,19,21,22,23,24,25,26,27,28,29,31,32,33,34,35,36] is reported in Table S1. This table contains information about the metabolites reported in each reference, such as their molecular formula, IDs (LipidMAPS and/or HMDB IDs), the extraction procedure performed, the analytical platform used, and the detected metabolite class. Readers can select metabolites dynamically by filtering data according to the latter information. A total of 1187, 111, and 128 metabolites were reported using LC-MS, GC-MS, and NMR, respectively (see Figure 4). As shown in the Venn diagram, LC-MS and GC-MS allowed the detection of 36 common metabolites (mainly carbohydrates and FAs); a total of 29 metabolites overlapped between LC-MS and NMR (principally oligosaccharides); and 21 metabolites (predominantly amino acids and organic acids) were commonly reported in GC-MS and NMR based studies. Only 13 metabolites were reported by all three platforms, i.e., creatine, tyrosine, arabinose, galactose, glucose, lactose, maltose, capric acid/caprate, caprylic acid/ caprylate, citric acid/citrate, pyruvic acid/pyruvate, hippuric acid/hippurate, and myo-inositol. These metabolites were assigned to different classes including amino acids, carbohydrates, FAs, and organic acids.

**Figure 5:**
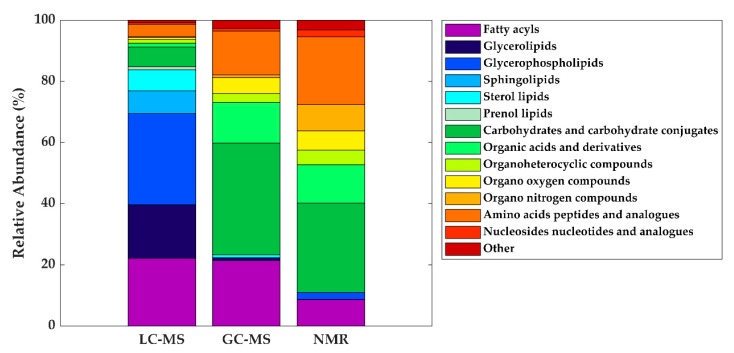
Distribution of metabolite classes annotated and/or identified in HM according to technique. Note: GC-MS, gas chromatography—mass spectrometry; LC-MS, liquid chromatography—mass spectrometry; NMR, nuclear magnetic resonance.

Based on the available data from the literature, the distribution of metabolite classes present in HM according to each technique was assessed. As can be seen in Figure 5, the difference in detected metabolite classes as observed by LC-MS in comparison to GC-MS and NMR is evident. Using GC-MS and NMR, carbohydrates are the most reported metabolites in HM, followed by amino acids, organic acids, organooxygen compounds, and organoheterocyclic compounds, with all these metabolite classes being certainly less abundant in LC-MS studies. In the case of NMR, organonitrogen compounds have also been reported, as well as nucleosides and nucleotides on a smaller scale. In the case of lipid classes, fatty acyls have been identified by LC-MS and GC-MS with similar incidence and in lesser extent by NMR. It is indubitable that lipid classes are more comprehensively studied by LC-MS assays, where glycerophospholipids, glycerolipids, and fatty acyls are detected at relatively high abundances, followed by sphingolipids, sterol lipids, and, to a lesser extent, prenol lipids.

Table 2 shows a list of metabolites reported in > 80% of studies employing either LC-MS, GC-MS, or NMR-based assays. This table is intended to aid method development of future untargeted metabolomics workflows tailored to the study of the HM metabolome, as it shows a shortlist of metabolites that should be detected by each platform regardless of the instrumental settings employed. It should be noted that due to the high versatility of LC-MS, there is a greater variation in metabolites recorded and in return, the list of consistently reported metabolites in HM across studies is shorter than for NMR and GC-MS, where differences in experimental conditions and variations between the employed detection parameters and instruments are smaller. Again, this table represents the high orthogonality between the detected metabolites using NMR and LC-MS. While the use of LC-MS is clearly of advantage for the measurement of different lipids, NMR provides information on amino acids and small organic acids. Metabolome coverage provided by GC-MS falls in-between the other two platforms, consistently providing information on lipids, sugars, amino acids, and organic acids.

## 6. Conclusions and Future Perspectives

In less than a decade, 26 research papers have been published trying to shed light on the complex and dynamic composition of HM and the feasibility of different options for sample extraction and metabolite detection has been demonstrated. Due to the many factors that influence HM composition, a thorough study design including SOPs for milk extraction, collection, and storage is indispensable for obtaining biologically meaningful results. Multi-platform approaches are encouraged for providing adequate metabolome coverage, as the diversity of compounds contained in HM will not be properly reflected using one single assay. In line with metabolomics workflows tailored to other sample types, the reproducibility of HM metabolomics studies will benefit from the implementation of QA/QC procedures. Automated metabolite annotation and identification with pure chemical standards is warranted and the authors encourage the use of publicly accessible platforms for enabling the exchange of raw data for comparison between studies.

## Figures and Tables

**Table 1:** Sample preparation steps and platforms employed in untargeted analysis of HM metabolome.

Sample Preparation (1st. step)	Sample Preparation (2nd. step)	Compound Class	Platform	Column/Capillary	References
Bligh & Dyer extraction	Deuterated solvent addition to aqueous phase	Polar metabolites	^1^H-NMR	-	[13,16,29,32]
	Derivatization of aqueous phase: methoximation and silylation	Polar metabolites and FAs	GC-MS	DB-5ms	[17,18,19]
	Derivatization of organic phase: methylation	FAs	GC-MS	DB-5ms	[13]
	Direct injection of aqueous phase	Polar metabolites	LC-QTOF-MS (+)	HILIC	[35]
	Redissolution of aqueous phase in H_2_O:ACN (95:5)	Polar metabolites	LC-Orbitrap-MS (+, −)	C18	[24]
	Redissolution of organic phase in (ACN:IPA:H_2_O (65:30:5)	Lipidic metabolites	LC-Orbitrap-MS (+,−)	C18	[25]
Folch extraction	Deuterated solvent addition to aqueous and organic phases	Hydrophobic and polar metabolites	^1^H-NMR	-	[28]
	Redissolution of aqueous phase in formic acid and centrifugation	Polar metabolites (amino acids)	CE-TOF-MS (+)	60 m × 50 µm I.D.	
	Redissolution of organic phase in IPA:H_2_O:ACN (2:1:1) and centrifugation	Lipidic metabolites	UPLC-QTOF-MS (+,−)	C18	
Single phase extraction	Derivatization: methoximation and silylation	Polar metabolites and FAs	GC-MS	DB-5ms	[27,28]
	Direct injection	Lipidic (and polar) metabolites	LC-QTOF-MS (+,−)	C8	[27,28]
			UPLC-QTOF-MS (+)	C18	[15]
Fat extraction with n-hexane/IPA	Deuterated solvent addition	TGs	^13^C-NMR; ^1^H-NMR	-	[20]
Filtration 3 kDa cutoff spin filter	Deuterated solvent addition	Polar metabolites	^1^H-NMR	-	[14,21,22,29,33]
Protein precipitation	Derivatization: methoximation and silylation	Polar metabolites	GC-MS	DB-5ms	[36]
	Hybrid SPE-Phospholipid extraction and redissolution in diluted organic phase of Bligh & Dyer extraction	Lipidic metabolites	LC-QTOF-MS (+)	C8	[35]
	Fat removal with CH_2_Cl_2_ and dansylation of aqueous phase	Polar metabolites (amine/phenol submetabolome)	Chemical isotope labelling LC-QTOF-MS (+)	C18	[45,46]
	Direct injection	Polar metabolites and FAs	UPLC-QTOF-MS (+,−)	C18	[18]
Fat removal by centrifugation	Two additional centrifugations and deuterated solvent addition	Polar metabolites	^1^H-NMR	-	[34]
	Filtration 10 kDa cutoff spin filter and deuterated solvent addition	Polar metabolites	^1^H-NMR	-	[23,26]
Homogenization	Deuterated solvent addition	Polar metabolites	^1^H-NMR	-	[31]
H_2_O-dilution	NaBH_4_-reduction and PGC cartridge	Oligosaccharides	UPLC-TQD-MS (+)	Hypercarb®	[24]

CE, capillary electrophoresis; FAs, fatty acids; GC, gas chromatography; HILIC, hydrophilic interaction liquid chromatography; IPA, 2-propanol; I.D., inner diameter; LC, liquid chromatography; MS, mass spectrometry; ^13^C-NMR, carbon-13 nuclear magnetic resonance; ^1^H-NMR, proton nuclear magnetic resonance; PGC, porous graphitic carbon; QTOF, quadrupole time of flight; TGs, triacylglycerols; TQD, triple quadrupole; UPLC, ultraperformance liquid chromatography; +, positive ionization mode; -, negative ionization mode.

**Table 2:** Most frequently reported metabolites (>80% of studies) according to technique.

Metabolite class	LC-MS	GC-MS	NMR
Fatty acyls	Linoleic acid (C18:2)	Oleic acid (C18:1)	-
	Oleic acid (C18:1)	Palmitic acid (C16:0)	
	Palmitoleic acid (C16:1)	Stearic acid (C18:0)	
Glycerolipids	DG (36:1)	-	-
Glycerophospholipids	LysoPC (16:0)	-	-
Carbohydrates and carbohydrate conjugates	-	Fructose	Lactose
		Fucose	
		Ribose	
Organic acids and derivatives	-	Malic acid Urea	Acetate
			Citrate
			Lactate
Organo nitrogen compounds	-	-	Choline
Amino acids, peptides, and analogues	-	Alanine Glutamate Glycine Pyroglutamic acid Serine Valine	Alanine Creatine Glutamate Glutamine Isoleucine Leucine Tyrosine Valine

GC-MS, gas chromatography—mass spectrometry; LC-MS, liquid chromatography—mass spectrometry; NMR, nuclear magnetic resonance; DG, diacylglycerol; PC, phosphatidylcholine.

## Supplementary Materials

The following are available online at https://www.mdpi.com/2218-1989/10/2/43/s1, Table S1: List of metabolites annotated and/or identified in HM metabolomic studies.
